# A microfluidic model of human dental pulp angiogenesis for preclinical drug and biomaterial testing

**DOI:** 10.1016/j.mtbio.2026.102776

**Published:** 2026-01-06

**Authors:** Sara Svanberg, Mathilde Hanoune, Thimios A. Mitsiadis, Petra S. Dittrich

**Affiliations:** a Department of Biosystems Science and Engineering, ETH Zurich, Switzerland; b Institute of Oral Biology, University of Zurich, Switzerland

**Keywords:** Angiogenesis, Dental pulp cells, HEMA, Limantrafin (CB-103), Paclitaxel, Emdogain, Dental pulp stem cells, “Organ-on-chip”, Biomaterials, Drugs, Dentistry, Regeneration, Endodontics

## Abstract

Dental pulp homeostasis and regeneration critically depend on angiogenesis, the formation of new capillaries from preexisting blood vessels. Regenerative endodontics has emerged as a clinical strategy to restore damaged or diseased dental pulp tissues using stem cells, signalling molecules, and scaffolds. Enhanced cell viability and angiogenesis are essential for the success of such regenerative therapies. However, their development, as well as efficient drug and biomaterial testing, is limited by the lack of physiologically relevant models. In this study, we developed a microfluidic model of angiogenesis in the human dental pulp to investigate the effects of drugs and biomaterials commonly used in dentistry. We optimised culture conditions influencing angiogenic sprouting and found that the presence of dental pulp cells and lower fibrin concentrations promoted angiogenesis significantly. Furthermore, static culture conditions enhanced sprouting compared with co- or contra-directional hydrostatic pressure-driven flow. Using this platform, we tested drugs (Paclitaxel and Limantrafin) and biomaterials (HEMA and Emdogain®). The model enabled quantitative imaging of angiogenic sprout growth and assessment of cytotoxicity through analysis of the culture medium. Importantly, the timing of drug exposure proved critical: early treatment inhibited sprout formation, whereas later treatment compromised the stability and viability of established vessels. HEMA (10 mM) resulted in cytotoxicity and compromised vessels, whereas Emdogain (1 mg/ml) showed no cytotoxicity and no significant impact on vessel formation. Paclitaxel efficiently inhibited angiogenesis at low concentrations (50 nM) with low cell death while Limantrafin required high concentrations (1 mM) to inhibit angiogenesis while showing elevated cell death and cytotoxicity. In conclusion, this microfluidic model provides a robust tool for studying fundamental angiogenic processes in the human dental pulp and offers an improved platform for safe and effective drug and biomaterial testing, thereby advancing regenerative endodontic therapies.

## Graphical abstract

**Figure:**
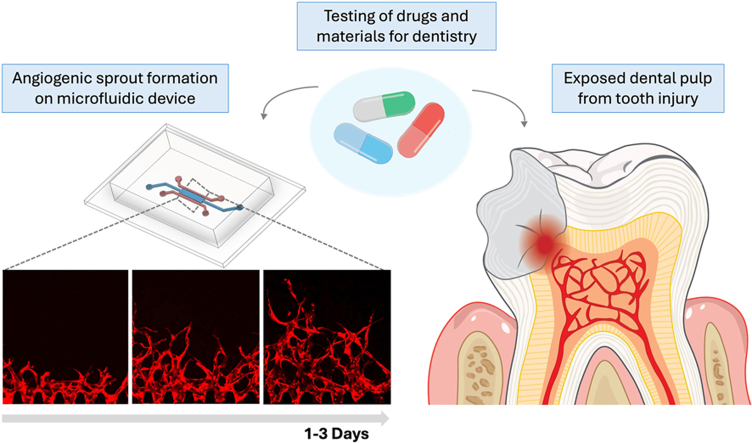

## 1 Introduction

The dental pulp is a specialized tissue that distinguishes itself from other craniofacial tissues through its extensive vascularization, despite being enclosed within a highly calcified environment. The vitality and homeostasis of dental pulp tissue rely on its abundant vasculature, which ensures proper exchange of nutrients, oxygen, and waste products [1]. However, complications affecting dental pulp physiology can arise from various pathological conditions or iatrogenic interventions, including trauma, infection, genetic diseases, and restorative dental procedures. Damage or infection of the dental pulp can lead to significant oral and systemic health complications, such as pulp necrosis, internal tooth resorption, and the generation of pulp stones, as well as cardiovascular and autoimmune diseases [2].

Restorative and regenerative procedures for the dental pulp aim to address tooth damage, infection, or decay while preserving tooth vitality and function. Regenerative endodontics has emerged in clinical dentistry as a strategy to restore the function of the damaged dental pulp-dentine complex using stem cells, scaffolds and signalling molecules, rather than replacing dental tissues with inert materials [3]. Importantly, maintaining cell viability and promoting the formation of new blood vessels through angiogenesis are critical for the success of both regenerative and restorative procedures.

Angiogenesis is defined as the formation of new capillaries from pre-existing blood vessels and is central to health, regeneration, and disease progression [4]. Moreover, angiogenesis is essential for understanding the metabolic, morphological, and functional responses of tissues to drugs and materials used in clinical practice and daily life. The cytotoxic response of the dental pulp to frequently used materials has been studied extensively. However, the testing of novel dental materials and oral-care products is still largely based on standards published by the International Organization for Standardization (ISO 10993), which typically rely on monolayer cultures of a single cell type [5]. These models are too simplistic to recapitulate human physiological responses. In recent years, in vitro three-dimensional (3D) organotypic models, known as “organ-on-chip” systems, have been introduced to better mimic the complex microenvironment of specific tissues. Such models also hold the potential to reduce reliance on animal testing, which often fails to reproduce human pathophysiological responses and contributes to costly and inefficient drug development [6].

To date, many organs and tissues have been modelled using microfluidic technologies, including the lung [7], gut [8,9], heart [10], and kidney [11]. In dentistry, such models have also gained increasing attention. For example, Franca et al. developed a model for material testing using dental pulp stem cells (DPSCs) and dentine extracts [12]. Subsequently, Rodrigues et al. presented a model incorporating a biomaterial-biofilm-dentine interface [13]. Models for studying infections were later introduced by Dhall et al. [14] and Makkar et al. [15]. More recently, Muniraj et al. developed a “gingiva-on-chip” model to investigate ulcers [16]. In our previous work, we established the first vascularized periodontal ligament model in the context of periodontitis [17]. However, none of these models have addressed angiogenesis in the dental pulp, which is a critical process for homeostasis and regeneration following dental treatment in this highly vascularized tissue.

Several models have been developed to study angiogenesis and its role in diseases such as cancer tumour growth [18] and systemic sclerosis [19] in related drug screenings [20]. However, models addressing angiogenesis in the context of dental organs for drug and material testing are still lacking. DPSCs have been identified as an attractive source of mesenchymal stem cells because of their accessibility and uncomplicated isolation. Their differentiation potential and proangiogenic properties make them highly promising for applications in tissue engineering and stem cell-based therapies [21]. The DPSCs can contribute to angiogenesis in two ways, either by differentiating into endothelial cells or by promoting endogenous angiogenesis, which both are critical processes in dental pulp regeneration [22].

To address the lack of relevant models for studying regeneration, angiogenesis, drug and material testing in endodontics and oral health, we developed a microfluidic model of angiogenesis in the human dental pulp. The microfluidic device was fabricated from polydimethylsiloxane (PDMS) and glass and designed as a three-channel system [23]. The central channel was seeded with patient-derived DPSCs embedded in a fibrin extracellular matrix. Human umbilical vein endothelial cells (HUVECs) were seeded in one of the side channels, from which they sprouted into the fibrin hydrogel. We first optimised the culture conditions by characterising factors influencing angiogenesis, including fibrin concentration, DPSC seeding density, and the presence of hydrostatic pressure-driven flow. We then tested clinically relevant drugs and biomaterials used in pulp capping and tissue regeneration, including 2-hydroxyethyl methacrylate (HEMA) and Emdogain®. In addition, we evaluated drugs known to affect angiogenesis, such as Limantrafin and Paclitaxel. Drug effects were evaluated by quantifying parameters related to angiogenic sprout growth and cell death through imaging, while cytotoxicity was assessed using the collected cell culture medium.

Our objective was to establish an angiogenesis model of the human dental pulp to evaluate the effects of tested drugs and biomaterials on cell death, cytotoxicity and angiogenesis. Overall, the development of angiogenesis models for the human dental pulp is essential for advancing regenerative endodontics and for enabling the testing of drugs and biomaterials to ensure that clinical interventions are both safe and effective.

## 2 Results

### 2.1 A microfluidic platform that supports angiogenic sprouting

**Fig. 1:**
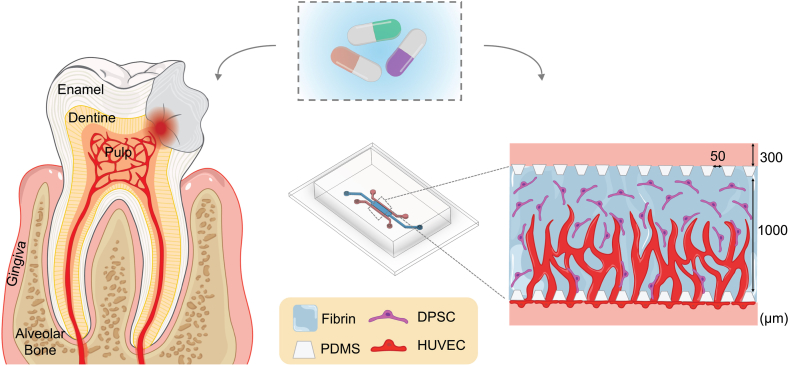
Sketch of a tooth injury where the highly vascularized dental pulp is exposed. Angiogenesis in the dental pulp is emulated on a three-channel microfluidic chip where dental pulp stem cells are seeded in fibrin hydrogel in the middle. HUVECs are seeded on the gel interface in the side channel, forming a monolayer that is sprouting into the gel. Here, the model is used for evaluating dentistry-related drugs and biomaterials.

To study angiogenesis in the dental pulp, we fabricated a microfluidic chip with three parallel channels separated by micropillars as described in our previous work [17]. The chip is casted in PDMS from a silicon wafer and subsequently bonded to a glass cover slide. The middle channel is filled with fibrin hydrogel loaded with DPSCs (Fig. 1). Thereafter, one side channel is seeded with HUVECs, and the chip is tilted at a 90° angle to let the HUVECs sediment and form a monolayer on the hydrogel interface. The chip is turned back to an upright position after 1h, and excess cells are washed away. Medium-filled pipette tips with equal volumes are placed in all inlets. The next day, angiogenic sprouts have spontaneously formed (SI Figure 1). If nothing else is mentioned, the medium is changed on day 3 and the cells are fixed and analysed on day 6. During the initial cultivation period, the medium is supplemented with vascular endothelial growth factor (VEGF, concentration: 50 ng/ml). We characterised the angiogenic sprouting by tuning fibrin and DPSC concentration and studied the effect of hydrostatic pressure-driven flow. Once optimal culture condition was achieved, the model was applied for drug and biomaterial testing.

### 2.2 The presence of dental pulp cells and lower fibrin concentration promotes angiogenic sprouting

**Fig. 2:**
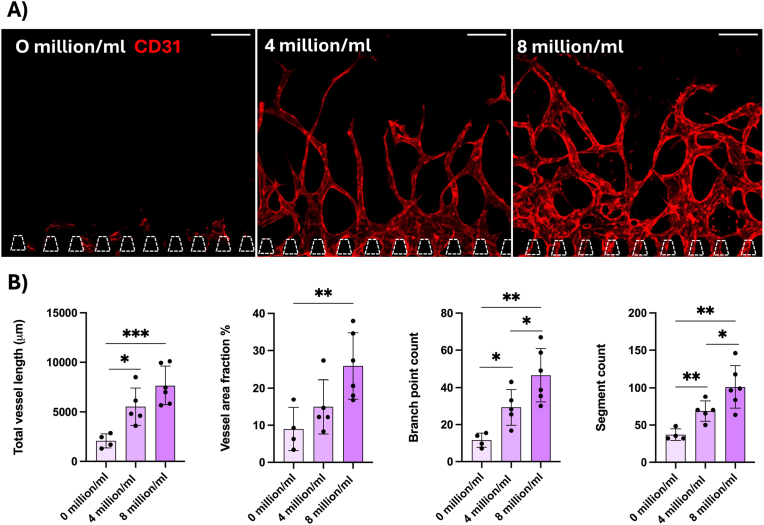
A) Angiogenic sprouts from HUVECs stained with CD31 (red) growing in fibrin hydrogel containing different concentrations of dental pulp stem cells. Scale bars: 200 μm. B). Quantification of total vessel length (μm), vessel area fraction (%), branch point count and segment count for the different dental pulp stem cell concentrations. Each data point represents one chip analysed in four distinct regions. Data presented as mean ± SD (∗p < 0.05, ∗∗p < 0.01, ∗∗∗p < 0.001). (For interpretation of the references to color in this figure legend, the reader is referred to the Web version of this article.)

In our system, we tested the influence of DPSCs on angiogenesis. We loaded the fibrin hydrogel with 0, 4 or 8 million cells/ml. Thereafter, we quantified several parameters, including total vessel length, vessel area fraction, branch point count and segment count using the REAVER program [24], a MATLAB-based program to analyse vascular network images. We found that angiogenic sprouting did not occur in the hydrogel in the absence of DPSCs (Fig. 2A). However, co-culture with 4 and 8 million cells/ml induced angiogenic sprouting. We found significantly more branching points and segments with higher DPSC concentration (Fig. 2B). These findings suggest that DPSCs are important for angiogenesis. Since the staining for platelet endothelial cell adhesion molecule 1 (PECAM-1, CD31), a widely used marker for endothelial cells, overlaps with the RFP signal from the HUVECs (SI Fig. 2), it indicates that the DPSCs mainly contribute to endogenic angiogenesis. We used 8 million DPSCs/ml in the following experiments.

**Fig. 3:**
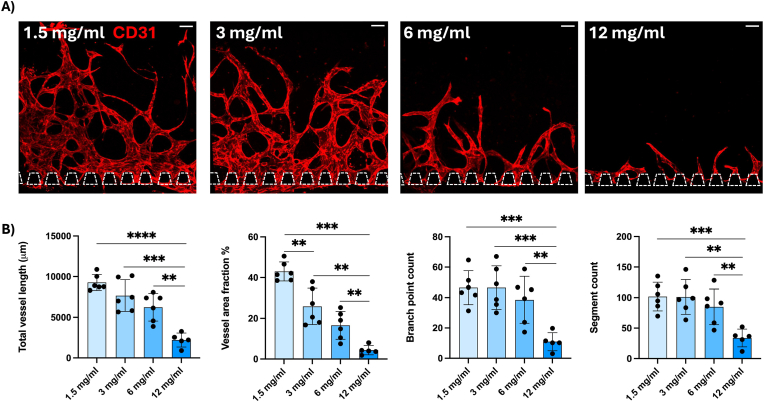
A) Angiogenic sprouts from HUVECs stained with CD31 (red) growing in hydrogels with different fibrin concentrations. Scale bars: 100 μm. B). Quantification of total vessel length (μm), vessel area fraction (%), branch point count and segment count. Each data point represents one chip analysed for three distinct regions. Data presented as mean ± SD (∗p < 0.05, ∗∗p < 0.01, ∗∗∗p < 0.001, ∗∗∗∗p < 0.0001). (For interpretation of the references to color in this figure legend, the reader is referred to the Web version of this article.)

Furthermore, we quantified the same growth parameters for different fibrin concentrations (1.5, 3.0, 6.0 and 12.0 mg/ml) and found that a lower fibrin concentration promotes angiogenic sprouting. The 3.0 and 6.0 mg/ml resulted in significantly more and longer sprouts compared to 12.0 mg/ml (Fig. 3). The lowest concentration (1.5 mg/ml) did result in more and longer sprouts than all higher concentrations; however, it was not always stable and reproducible as it sometimes detached or broke (SI Fig. 3). Therefore, we continued the following experiments with a concentration of 3 mg/ml.

### 2.3 Hydrostatic pressure-driven flow does not improve the angiogenic sprouting

**Fig. 4:**
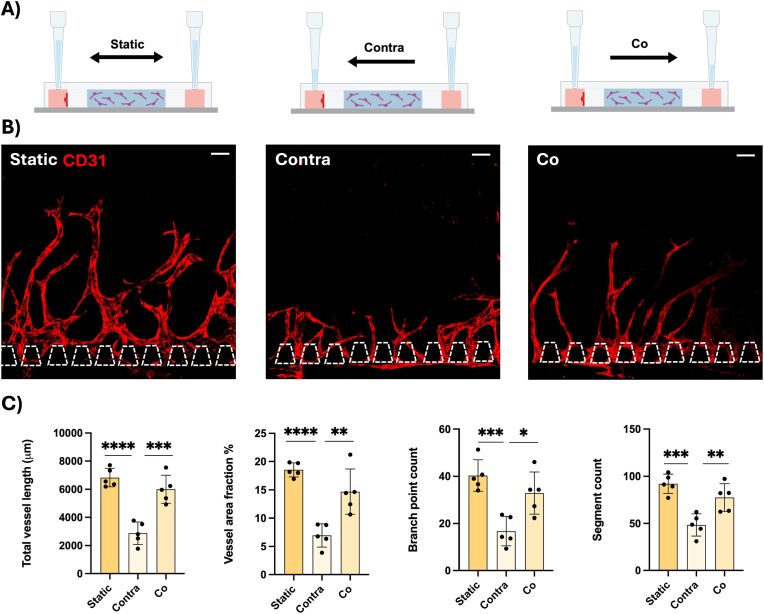
A) Flow generation driven by hydrostatic pressure from using pipette tips containing different volumes. B) Angiogenic sprouts of HUVECs stained with CD31 (red) growing during static (left), contra-directional (middle) or co-directional flow (right) conditions. Scale bars: 100 μm. C). Quantification of total vessel length (μm), vessel area fraction (%), branch point count and segment count for the different hydrostatic pressures. Each data point represents one chip analysed for four distinct regions. Data presented as mean ± SD (∗p < 0.05, ∗∗p < 0.01, ∗∗∗p < 0.001, ∗∗∗∗p < 0.0001). (For interpretation of the references to color in this figure legend, the reader is referred to the Web version of this article.)

In our system, we tested the influence of hydrostatic pressure-driven flow, which was generated through different volumes of medium in the pipette tips (Fig. 4a). The hydrostatic pressure-driven flow was generated either in the growing direction of the angiogenic sprouts (co-directional) or in the opposite direction (contra-directional). These were compared to no hydrostatic pressure-driven flow (static), where the volumes in the pipette tips were equal. The static condition resulted in more and longer angiogenic sprouts than the hydrostatic pressure-driven flows. Contra-directional flow resulted in significantly fewer and shorter sprouts. The co-directional flow did not significantly differ from the static condition (Fig. 4b and c). Given the influence of the tested parameters on angiogenic sprouting, we chose to use 8.0 million cells/ml DPSCs in 3 mg/ml fibrin, cultivated under static conditions for the following experiments.

### 2.4 Characterisation of vessels’ quality and their growth on the chip over time

**Fig. 5:**
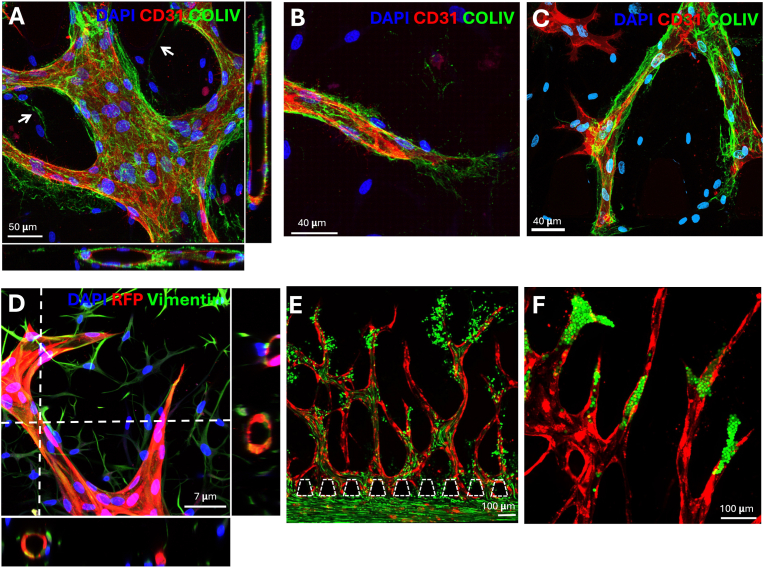
Maximum intensity projections of confocal z-stacks of angiogenic sprouts (A–D). A) Cross-section of angiogenic sprouts of HUVECs stained with CD31 (red), collagen IV (green) and dapi (blue). White arrows indicate collagen IV production by dental pulp stem cells. Scale bar: 50 μm. B) and C) angiogenic tip cells stained with CD31 (red), collagen IV (green) and dapi (blue). Scale bar: 40 μm. D) Cross-section of angiogenic sprouts with red fluorescent protein (RFP)-producing HUVECs (red) and dental pulp stem cells stained with vimentin (green). Scale bar: 7 μm. E) Angiogenic sprouts perfused with 1 μm fluorescent beads (green). Scale bar: 100 μm. F) Angiogenic sprouts perfused with 10 μm fluorescent beads (green). Scale bar: 100 μm. (For interpretation of the references to color in this figure legend, the reader is referred to the Web version of this article.)

Next, we characterised the vessels’ quality and their growth on chip over time. We first used high-resolution imaging to show cross-sections of the vessels that form well-defined lumens (Fig. 5 a and d) where the endothelial cells are lining a collagen IV basement membrane. The DPSCs were stained with vimentin and showed elongated spindle-like morphology (Fig. 5 d). The DPSCs were also positive for alpha smooth muscle actin (αSMA) while aligning along the vessels (SI Fig. 5). The DPSCs produce collagen IV (Fig. 5a, indicated by white arrows) between vessels which could be facilitating anastomosis. Tip cells guiding the angiogenesis was also observed (Fig. 5 c). We also showed that the vessels are perfusable with fluorescent beads. Smaller beads with a 1 μm diameter accumulated at the end of the vessel and eventually could leak (Fig. 5 e). Beads with a 10 μm diameter also accumulated at the tips or narrow sections, but they were too large to leak (Fig. 5 f). Overall, the model has vessels with well-formed and perfusable lumens and cells expressing relevant physiological markers.

**Fig. 6:**
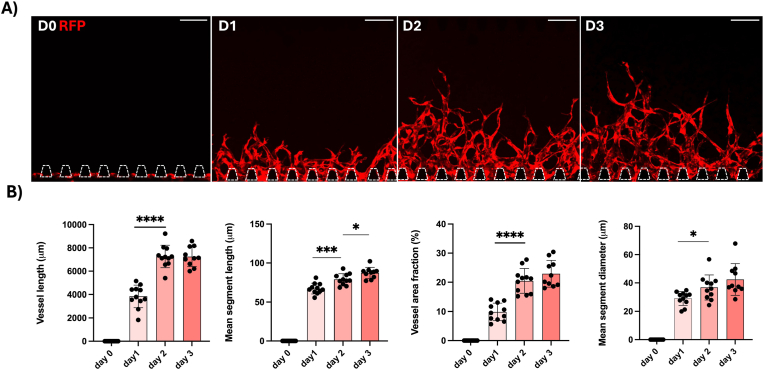
A) Maximum projections of confocal z-stacks showing growth of angiogenic sprouts from red fluorescent protein (RFP) producing HUVECs (red) monitored live over 3 days. Scale bars: 200 μm. B) Quantification of total vessel length (μm), mean segment length (μm), vessel area fraction (%), mean segment diameter (μm). Each data point represents one chip analysed for four distinct regions. Data presented as mean ± SD (∗p < 0.05, ∗∗p < 0.01, ∗∗∗p < 0.001, ∗∗∗∗p < 0.0001). (For interpretation of the references to color in this figure legend, the reader is referred to the Web version of this article.)

Next, we characterized the growth on chip to determine suitable treatment intervals for the drug and biomaterial testing experiments. The cells were seeded on the chip on day 0 and the growth of angiogenic sprouts was monitored for 6 days by means of the red fluorescent protein (RFP) producing HUVECs. We found that the vessel growth is most striking between days 1 and 2 (Fig. 6). Therefore, treatment on day 1 will reveal immediate effects on initial angiogenesis, while treatment on day 3 shows the impact on more advanced angiogenic sprouting. Thereby, this model enables us to study the effect on forming as well as on existing vessels, which co-exist in tissues in vivo. Additionally, a few chips were kept in static culture for a longer time to study the development of the vessels’ morphology (SI Fig. 4), which showed that the sprouts reached the opposite side channel by day 9, after which the vessel coverage area and the vessel diameter decreased over time.

### 2.5 Angiogenesis model for drug and biomaterial testing

**Fig. 7:**
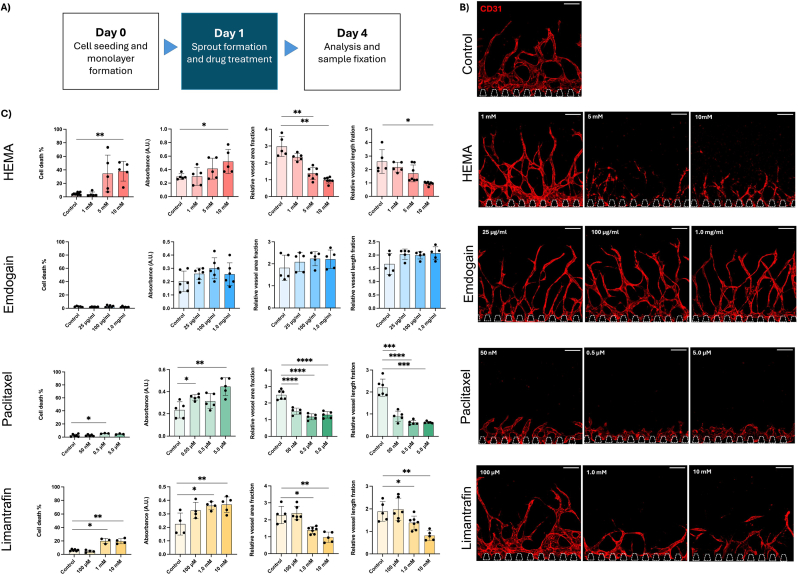
A) Schematic sketch of treatment schedule for day 1. B) Maximum projections of confocal stacks of HUVECs (red, CD31) treated on day 1 and fixed on day 4. Scale bars: 200 μm. C) Quantification of cell death %, LDH release and fold increase in vessel area fraction and vessel length after treatment. Each data point represents one chip analysed for three distinct regions. Data presented as mean ± SD (∗p < 0.05, ∗∗p < 0.01, ∗∗∗p < 0.001, ∗∗∗∗p < 0.0001). (For interpretation of the references to color in this figure legend, the reader is referred to the Web version of this article.)

**Fig. 8:**
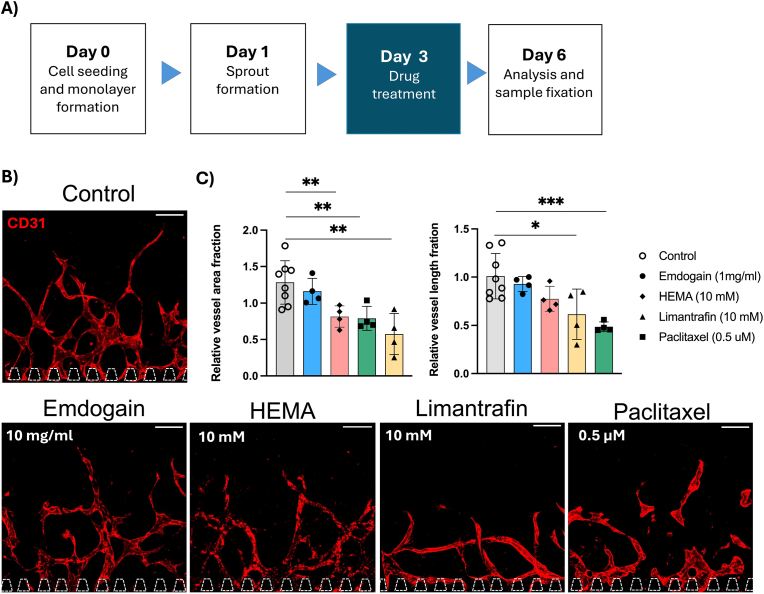
A) Schematic sketch of treatment schedule for day 3. B) Maximum projections of confocal stacks of HUVECs (red, CD31) treated on day 3 and fixed on day 6. Scale bars: 200 μm. C) Quantification of cell death (in %), LDH release, fold increase in vessel area fraction and vessel length after treatment. Each data point represents one chip analysed for three distinct regions. Data presented as mean ± SD (∗p < 0.05, ∗∗p < 0.01, ∗∗∗p < 0.001, ∗∗∗∗p < 0.0001). (For interpretation of the references to color in this figure legend, the reader is referred to the Web version of this article.)

HEMA is a resin monomer used as a gold standard in dentistry to restore damaged teeth. However, if not properly polymerized, it can leak into the pulp tissue and exert cytotoxic effects [25]. We studied the impact of HEMA on angiogenesis by treating our chips with HEMA dissolved in culture medium (1, 5, and 10 mM) either on day 1 (Fig. 7) or day 3 (Fig. 8). After 72 h of treatment, the supernatant was collected for LDH analysis to assess cell cytotoxicity. Additionally, the percentage of dead cells was determined (SI Fig. 6). The chips were then fixed, stained and imaged to quantify the total vessel length and the vessel area fraction using the REAVER program.

When treated on day 1, angiogenesis was significantly reduced for 5 and 10 mM compared to 1 mM HEMA or untreated. The LDH release and cell death were significantly upregulated at 10 mM, but not for the lower concentrations (Fig. 7C). Given the significant effect of 10 mM HEMA on angiogenic sprouting, we also tested this concentration for treatment on day 3, where the angiogenic sprouting was more advanced (Fig. 8). Treatment on day 3 also resulted in significant increase of dead cells and LDH release as well as decreased vessel area fraction, but a less dramatic effect on vessel area fraction and total vessel length (Fig. 7, Fig. 8c, SI Fig. 8).

Emdogain is also a commonly used product in dental clinics to restore the tooth supporting tissues. It is extracted from developing pig teeth and contains a mixture of active ingredients including enamel matrix proteins [26]. Here, we investigated its effect on angiogenesis. Our experiments did not show any significant increase in vessel area fraction or total vessel length, even though the trend is slightly increased compared to the control for all used concentrations (25 μg/ml, 100 μg/ml and 1 mg/ml). Furthermore, we did not observe any cytotoxic effect or increase in cell death, suggesting it is a safe drug. When administered on day 3, on more advanced angiogenic sprouts, we observed the same trends.

In addition to dentistry-related drugs and biomaterials, we further applied the model to test drugs targeting angiogenesis, Paclitaxel and Limantrafin. Paclitaxel is one of the first anti-cancer drugs having been developed to target mitosis in many different cancer and tissue types. In our model, concentrations as low as 50 nM inhibited angiogenesis significantly (Fig. 7, SI Fig. 7), while no angiogenesis took place from 0.5 μM. The morphology of the vessel was drastically changed with round and bulky vessels after treatment. LDH release showed cytotoxic effect at 50 nM, but the cell death showed a significant increase only at 0.5 μM. Given the critical effect on angiogenic sprouting at 0.5 μM, we also investigated this concentration on day 3 when angiogenic sprouts were more advanced. Interestingly, Paclitaxel also affects more advanced angiogenic sprouts, which was confirmed with significantly decreased vessel area fraction, total vessel length and morphological change after treated on day 3. Similarly to the treatment on day 1, the cell death was significantly increased but not the LDH release (SI Fig. 8). Limantrafin is a NOTCH inhibitor that blocks an important pathway in angiogenesis and is currently in clinical trial. Limantrafin decreased angiogenesis only at a high concentration (1 and 10 mM), both of which concentrations exhibited a significant increase in cell death and LDH release (Fig. 7). Treatment with 10 mM limantrafin on day 3 resulted in the same trend as when treated on day 1 (SI Fig. 8).

## 3 Discussion

Since DPSCs were first isolated and characterized in 2000, they have been regarded as a promising cell source for regenerative therapies in dentistry and beyond, owing to their differentiation potential and minimally invasive collection [27]. This has been supported by subsequent studies investigating the application of DPSCs in tissue engineering and highlighting their proangiogenic potential [[28], [29], [30], [31]]. In this work, we present the first microfluidic model of angiogenesis in the dental pulp for oral health-related drug and biomaterial testing. Compared with previous angiogenesis models, our approach incorporates patient-derived DPSCs to support vessel formation, and we demonstrate that DPSCs play a critical role in angiogenesis. The presented microfluidic platform offers many advantages over traditional well plate-based or hydrogel systems. In addition to controlling medium handling and to enabling studies on hydrostatic pressure-driven flow, the microfluidic approach reduces consumption of cells and drugs that are often scarce and expensive. Furthermore, this model provides ideal imaging conditions for efficient quantification and analysis of angiogenic sprouting as the angiogenic sprouts grow in a defined and reproducible manner, determined by the device geometry. Additionally, the thin glass cover slip and the tissue thickness enable high resolution imaging.

Angiogenesis is initiated by various factors, including growth factors, cytokines, matrix metalloproteinases (MMPs), adhesion molecules, insufficient oxygen and nutrient supply, and components of the extracellular matrix (ECM) [4]. DPSCs consume oxygen and nutrients and remodel the microenvironment by producing ECM proteins such as fibronectin, laminin, and collagen [32]. For example, we observed that the DPSCs produce collagen IV (Fig. 6 and SI Fig. 5), forming bridges between angiogenic sprouts which may contribute to the anastomosis. Interestingly, we also found that DPSCs are positive for αSMA and align along vessels in a spindle-shaped morphology. αSMA is a cytoskeletal protein involved in contractile functions and is expressed in vascular smooth muscle cells and pericytes, where it contributes to vascular development [33]. αSMA-positive cells have also been reported in immature dental pulp and periodontal ligament tissues in proximity to blood vessels [34]. We propose that DPSCs support vessel formation, stabilization, maturation, and remodelling, which is similar to the role of pericytes and consistent with our previous observations in patient-derived periodontal ligament cells [17]. Given that the CD31 marker was overlapping with the RFP signal of the HUVECs, it suggests that the DPSCs support the angiogenesis in an endogenous manner. In this context, Pagella et al. demonstrated that DPSCs and PDLSCs share similar molecular signatures despite residing in different microenvironments [35]. Additional staining confirmed the presence of markers such as CD31, ZO-1, and vimentin, as well as the perfusability of sprouts. In summary, we have established a viable angiogenesis model for the dental pulp.

Our model is also suitable for studying culture conditions, such as matrix properties and the effects of hydrostatic pressure-driven flow. We employed a fibrin matrix within the physiological stiffness range of soft tissues (0.1–30 kPa), corresponding to fibrin concentrations of 2–20 mg/ml [36]. We observed that lower fibrin concentrations enhanced angiogenesis, likely due to reduced resistance to cell migration.

In the body, blood flow exerts shear stress on endothelial cells and thereby influences vascular remodelling. Furthermore, interstitial flow arises from pressure differences between ECM and the circulatory and lymph systems [37]. The impact of shear stress on angiogenesis remains controversial, as studies have reported both attenuating and promoting effects on sprouting [[37], [38], [39]]. In our previous studies, where vascular networks were generated through “vasculogenesis-on-chip”, vessel diameter, morphology, and permeability were significantly altered by introducing peristaltic or intestinal flow compared with static conditions [17,23]. In contrast, in the present study we found that static culture conditions resulted in greater angiogenic sprouting. The key difference between the two systems is that, in “vasculogenesis-on-chip”, endothelial cells were mixed with dental pulp cells or fibroblasts in the hydrogel, whereas in the angiogenesis model, endothelial cells were introduced only in the side channel to sprout into the hydrogel containing DPSCs. It is possible that paracrine factors become more critical in the later described configuration and accumulate more effectively under static conditions. Moreover, angiogenesis is known to be triggered by hypoxia, nutrient deprivation, and low shear stress, all of which are indicative of insufficient blood supply. We therefore propose that a combination of these factors contributed to the increased number and length of angiogenic sprouts observed under static conditions.

Once optimal culture conditions for generating angiogenic sprouts had been established, we applied the model for drug and biomaterial testing. We selected compounds relevant to different aspects of oral health. Composite resins are widely used for restoring damaged teeth, and HEMA is one of the most common resin monomers. However, when these cytotoxic monomers are not fully polymerized, they can diffuse into the dental pulp and cause cytotoxicity [[40], [41], [42]]. In our model, HEMA affected both cell viability and vessel formation at concentrations starting from 10 mM, in agreement with previous findings [25]. The actual in vivo concentration depends on factors such as dentine thickness, adhesive type and amount, curing efficiency, and pulp blood flow, which can dilute monomer leakage. Dentine disc diffusion models have reported concentrations of 370–449 nmol after 24 h [42,43], corresponding to 0.37-0,45 mM in 1 ml of eluate. Although these values are lower than the effective concentrations observed in our model, studies have demonstrated that long-term exposure to subtoxic levels of HEMA can still impair the differentiation and mineralisation of DPSCs [44].

Emdogain, a dental regenerative product used clinically to restore tooth supporting tissues, did not induce cytotoxic effects or cell death in our model. Although we observed a slight increase in vessel area fraction and total vessel length, these changes were not statistically significant. For experimental convenience, we diluted the viscous Emdogain solution to 1 mg/ml, compared with the 30 mg/ml concentration typically applied in clinics. However, in vivo the material is spread into a thin layer and subject to dilution by saliva and tissue diffusion. Moreover, DPSCs are known to secret angiogenic factors [30,45,46], which may have masked or diminished the contribution of Emdogain.

Angiogenesis is not only essential for maintaining healthy tissues but also drives pathological processes such as tumour growth during cancer. Oral squamous cell carcinoma (OSCC), which accounts for 90 % of head and neck cancers, is the seventh most common cancer worldwide [47,48]. Consequently, many cancer therapies use drugs targeting angiogenesis. Paclitaxel is among the first anti-cancer drugs to have been developed and is now approved for a broad clinical use, including breast, lung, and ovarian cancer. Paclitaxel acts on the cytoskeleton by stabilising the microtubules and thereby preventing cell division [49]. In addition, several studies have reported anti-angiogenic effects of Paclitaxel [[50], [51], [52]]. Our experiments confirmed Paclitaxel as a potent anti-angiogenesis drug. Notably, we observed a strong effect at already concentrations as low as 50 nM, which is considerably lower than in other studies that reported anti-angiogenic activity only at 5.0 μM[53]. This discrepancy may arise from differences in supporting cell types: in our model, DPSCs were used instead of fibroblasts to promote vascular formation. The dental cell types may be more sensitive or exhibit a higher proliferation rate, making them more susceptible to anti-mitotic drugs such as paclitaxel. These findings underscore the importance of including tissue-specific cells in oral health for drug screening and highlight the relevance of considering oral health in cancer patients undergoing chemotherapy.

In contrast to Paclitaxel, Limantrafin (CB-103) is an experimental anti-cancer drug that has not yet been approved for clinical use and is currently undergoing preclinical and early clinical trial [48]. It is designed to inhibit the NOTCH signalling pathway, a key regulator of angiogenesis [[54], [55], [56]]. In our study, high concentrations (1 and 10 mM) were required to achieve an inhibitory effect on angiogenesis, while exhibiting cell cytotoxicity. In comparison, previous studies performed in 2D cultures reported effective inhibition of the NOTCH pathway in the micromolar range [57]. These findings suggest that, within our 3D vascularized system, considerably higher concentrations of Limantrafin were needed to observe an effect, possibly due to factors such as higher cell density and drug absorption by the hydrogel matrix and PDMS. In this regard, paclitaxel demonstrated greater potential as an efficient angiogenesis inhibitor in our 3D vascularized model. More broadly, this highlights the importance of evaluating drug efficacy in complex 3D systems, as effective concentrations can differ substantially from those observed in traditional monolayer cultures.

In this study, our focus was on angiogenesis and its response to different culture conditions and drugs or biomaterials. We used HUVEC cells as they are easily available, robust, reproducible, and well characterized. However, they are not tissue specific to the dental pulp and can possibly introduce physiological and functional mismatches relative to native dental pulp vasculature. In addition to tissue specific-endothelial cells, it would also be valuable to integrate additional cell types, such as immune cells and nerve cells, which play crucial roles in dental pulp homeostasis. Other future improvements include modifications of the hydrogel to resemble the extracellular matrix more precisely as well as the integration of a dentine interface, as many drugs and materials must diffuse through this layer to reach the dental pulp when the tooth is intact and healthy. However, engineering a dentine layer remains challenging; therefore, dentine extracts have been used as an alternative in previous studies [12]. The current model could also be applied to study fundamental disease mechanisms or regenerative biology, as proteomic analyses can be performed by adapting protocols previously established for similar microfluidic chips [58]. Future efforts could also explore new combinatorial drug treatments to enhance clinical outcomes and evaluate effects on oral tissues that may be indirectly impacted.

## 4 Conclusion

Overall, in this work we presented a microfluidic model of angiogenesis in the human dental pulp for drug and biomaterial testing with potential for broad applications. The model is easy to produce and handle, requiring only small sample volumes. Its compatibility with direct imaging and collection of culture medium provides a significant advantage over other platforms, such as transwell systems. We propose that this model can serve as a steppingstone for preclinical drug testing, offering early indications of the efficacy and safety of drug candidates and biomaterials before advancing to animal studies and clinical trials.

## 5 Materials and methods

**Cell culture**: The procedure for the collection of anonymised human dental pulp stem cells (DPSCs) at the Centre of Dental Medicine (ZZM) of the University of Zurich was approved by the Ethic Commission of the Kanton of Zurich (reference number 2012-0588) and the patients gave their written informed consent. The teeth were extracted by dentists at the clinic of Cranio-Maxillofacial and Oral Surgery Department at the Centre of Dental Medicine of the University of Zurich according to previously described protocols [[59], [60], [61]]. The stem cell potential of DPSCs was showed through osteogenic, adipogenic and neurogenic differentiation and through immunostainings using stem cell markers in previous studies [61,62]. The DPSCs were cryopreserved for future experiments [[60], [61], [62]]. The DPSCs were cultured to 90 % confluency in T75 culture flasks (VWR International GmbH, 391–3146) with Dulbecco's Modified Eagle Medium/Nutrient Mixture F-12 Glutamax medium (DMEM F-12 Glutamax, Thermo Fisher) supplemented with 10 % Fetal bovine serum (FBS, Gibco) and 1 % penicillin/streptomycin (Thermo Fisher). Red fluorescent protein (RFP) human umbilical vein endothelial cells (HUVECs) (Angio Proteomie) were cultured to 90 % confluency in endothelial growth medium (Vasculife, Cell systems) in T75 culture flasks precoated with 0.2 % gelatine (Sigma) for 1 h at 37 °C. All cells were cultured at 37 °C, 5 % CO_2_ and 95 % humidity and used up to passage 6–7. The medium was changed every two days.

**Fabrication of microfluidic chip**: PDMS was prepared by mixing the polymer (PDMS, Sylgard 184) and curing agent (Sylgard 184) at 10:1 wt% ratio followed by a degassing step and subsequently poured on a silicon master mold followed by a curing step in an oven at 80 °C for 3 h. The master mold was fabricated using standard lithography methods previously described [17]. The cured PDMS slab was cut into individual chips and all inlets were punched with a 1 mm biopsy punch (Miltex). The structured surfaces were cleaned with scotch tape and placed in a plasma oven (Harrick Plasma PDC-32G) facing upwards along with a cover glass (24x40 #1.5, Novoglas) and were surface treated at 50 W for 30 s. After treatment, the structured side was bonded to the glass slide. The chips were stored overnight in 80 °C to recover hydrophobicity. Finally, the chips were UV treated for 30 min and stored in a sterile Petri dish until further use.

**Cell seeding on microfluidic chip**: All cell types were grown to 90 % confluency. Then they were washed with phosphate buffered saline (PBS(−), Gibco) and then detached by adding 1 ml 0.05 % trypsin–ethylenediaminetetraacetic acid (trypsin-EDTA, Gibco) and incubated at 37 °C, 5 % CO_2_ for 5 min. Thereafter, the trypsin was inactivated by adding 10 ml of respective cell culture medium and transferred into 15 ml falcon tubes and centrifuged at 1500 rpm for 5 min. The supernatant was removed, and each cell pellet was resuspended in their respective cell culture medium followed by cell counting. A volume corresponding to 120 000 DPSCs were pipetted into an Eppendorf tube and centrifuged at 1500 rpm for 5 min. The supernatant was removed, and the cell pellet was resuspended and thoroughly mixed in 15 μl fibrinogen (1.5, 3.0, 6.0 or 12 mg/ml, Merck) resulting in a cell concentration of approximately 8 million cells/ml. Thrombin from human plasma (Merck) was diluted to 10 U/ml in DMEM F-12 Glutamax and 1.5 μl of this mixture was added to the cell-fibrinogen mix. 10 μl of this cell-hydrogel mix was immediately pipetted into the middle channel of the microfluidic 3-channel chip. Thereafter, the seeded microfluidic chips were placed in a petri dish containing a smaller Petri dish filled with PBS to maintain humidity while being incubated at 37 °C, 5 % CO_2_, for 15 min to let the hydrogel fully polymerize. Then the side channels were filled with medium and incubated for 1 h at 37 °C, 5 % CO_2_. Next, HUVECs were detached and counted in the same manner as previously described. One side of the microfluidic chips was emptied and replaced with a solution of 10 million cells/ml HUVECs. The chip was then incubated for 1 h with a 90° tilt to promote cell adhesion on the hydrogel interface. Thereafter, the chips were flipped back to original orientation and the culture medium in the side channels was replaced with VEGF-supplemented Vasculife medium (50 ng/ml, Rec Human VEGF, Gibco). The medium was not changed during the 3 first days to ensure accumulation of cell-secreted factors that promote HUVECs to sprout into the hydrogel.

**Dental biomaterials and angiogenic compounds**: 2-hydroxyethy methacrylate (HEMA, Sigma-Aldrich) was diluted in Vasculife medium to final working concentration of 1, 5, and 10 mM. The HEMA solution was sterile filtered (0.2 μm) before use. Emdogain Gel 30 mg/ml (Straumann) was diluted in Vasculife to a final working concentration of 25 μg/ml, 100 μg/ml and 1.0 mg/ml. Limantrafin (MCE, HY-135145) was reconstituted in Dimethyl sulfoxide (DMSO) (Huberlab, A3672.0100) to 100 mM and was then diluted in Vasculife medium to final working concentrations of 100 μM, 1.0 mM and 10 mM. Paclitaxel (Sigma-Aldrich, T7191) was diluted in Vasculife to a final working concentration of 0.05, 0.5 and 5.0 μM. The drug and material solutions were added on the chips in both side channels on either day 1 or day 3 and was analysed on day 4 and 6, respectively.

**REAVER analysis**: The chips were imaged using Nikon Ti2 spinning disk (Yokogawa) confocal microscope (Visitron) with a 10X objective. Z-stacks were acquired with 2 μm step size with a total thickness of 70 μm. Each chip was imaged for at least three distinct regions of interest (ROIs). ImageJ was used to process the stacks. A maximum projection was created of each stack followed by adjusting and cropping a field of view (950 × 1100 μm). The projections were segmented in Rapid Editable Analysis of Vessel Elements Routine (REAVER), a MATLAB based code [24], to generate quantitative parameters of the angiogenic sprouts including vessel area fraction, total vessel length, branchpoint count and segment count.

**Cell viability testing**: Cells on the chips were stained with Nucblue (DAPI, live cells) and Draq7 (dead cells) live to determine cell death ratio. Detailed dyes are listed in Table 1. The stains were diluted in Vasculife medium and incubated on the chips for 30 min at 37 °C, 5 % CO_2_ followed by as washing step with Vasculife medium. The chips were then imaged using Nikon Ti2 spinning disk (Yokogawa) confocal microscope (Visitron) with a 20X objective (SI Fig. 5). Z-stacks were acquired with 2 μm step size with a total thickness of 70 μm. Each chip was imaged for at least three distinct ROIs. ImageJ was used to process the stacks. A maximum projection was created of each stack and a mask, and a macro was created for the nucleus and dead stain channels. Cell death percentage was calculated using the following equation, where N is the number of cells:

$$Cell\;death\;\%=\frac{N_{Draq7}*100}{N_{Nucblue}}$$

**Table 1:** List of antibodies and dyes.

Reagents	Host	Dilution	Supplier	Catalog/Reference #
***Conjugated antibodies***				
*Anti*-aSMA AlexaFluor 640	Mouse	1:100	Invitrogen	50-9760-82
*Anti*-Hu Collagen IV AlexaFluor 640	Mouse	1:100	eBioscience	52-9871-82
*Anti*-Hu CD31 (PECAM-1) FITCH	Mouse	1:100	Invitrogen	*11-0319-42*
***Stainings***				
Nucblue (DAPI)	N/A	1:1000	Thermo Fisher	R37605
Draq7 (Dead)	N/A	1:1000	Thermo Fisher	D15106

**Cell cytotoxicity (LDH) testing**: To assess the cytotoxic effect, the supernatants of the treated chips were collected and analysed in a colorimetric lactate dehydrogenase (LDH) cytotoxicity assay used according to manufacturer instructions (Roche). LDH is a cytosolic enzyme present in the cytosol of many cells. If the plasma membrane is damaged due to toxic compounds, LDH is released in the surrounding cell culture medium, and can be quantified through a coupled enzymatic reaction in a colorimetric assay. In brief, supernatants were added 100 μl/well in a 96-flat bottom well plate in triplicate per chip. Culture medium was used as controls. Freshly prepared Reaction mixture (100 μl) was added to each well and incubated for 30 min at room temperature. Subsequently, stop solution (50 μl) was added to each well. The colorimetric output was immediately analysed in a plate reader (BioTek) at 490 nm and at a reference wavelength at 680 nm. Absorbance at 680 nm was subtracted from 490 nm. Furthermore, the average background from the medium control was subtracted from each sample. The resulting absorbance for each sample was finally plotted.

**High resolution imaging and cross-section generation**: After fixing and staining, the chips were imaged with a confocal microscope (Zeiss LSM 980 Airyscan 2) where the images were subsequently airyscan processed. Z-stacks were acquired with a 32X objective (zoom 1.3), 0.5 μm stepping size and a thickness of 60 μm. The stacks were further processed in Imaris software version 9.5 to create cross-sections and 3D constructions of the angiogenic sprouts.

**Immunohistochemistry**: The chips were fixed using 4 % paraformaldehyde (PFA, Thermo Fisher) for 30 min at room temperature followed by washing 3 times with PBS(−). Thereafter, the chips were permeabilized with 0.2 % tritonX (Sigma) in PBS for 30 min at room temperature followed by a blocking step with 0.1 % tritonX and 10 % goat serum (Thermo Fisher) for 1h at room temperature. Then the chips were incubated with antibodies and Nucblue diluted in 0.1 % triton and 1.5 % bovine serum albumin (BSA, Sigma) in PBS (−) overnight in fridge at 4 °C. Then the chips were washed with PBS(−) at least three times. Detailed antibodies and stains are listed in Table 1.

**Statistical Analysis and plotting**: All experiments were performed as at least triplicates and at least three ROIs per chip were used to create mean values per chip used for data and image analysis. All data was tested for normality (QQ plot) followed by a Welch's *t*-test. The data is represented as mean ± standard deviation (SD) if nothing else is mentioned. Significant difference was considered if p < 0.05. All plotting and statistical analysis was performed using GraphPad prism 9.5.1 for Mac (GraphPad Software, San Diego, California, USA).

## CRediT authorship contribution statement

**Sara Svanberg:** Writing – original draft, Methodology, Investigation, Data curation, Conceptualization. **Mathilde Hanoune:** Investigation. **Thimios A. Mitsiadis:** Writing – review & editing, Funding acquisition. **Petra S. Dittrich:** Writing – review & editing, Supervision, Funding acquisition, Conceptualization.

## Data availability statement

The data that support the findings of this study are available from the corresponding author upon reasonable request.

## Declaration of competing interest

The authors declare that they have no known competing financial interests or personal relationships that could have appeared to influence the work reported in this paper.

## Data Availability

Data will be made available on request.
