# Supplementary material for: A microfluidic model of human dental pulp angiogenesis for preclinical drug and biomaterial testing
Source: Mater Today Bio. 2026 Jan 6;37:102776. doi: 10.1016/j.mtbio.2026.102776 (PMC12830287; doi:10.1016/j.mtbio.2026.102776)
Supplement: Multimedia component 1 [file mmc1.pdf]

Supplementary information

A microfluidic model of human dental pulp angiogenesis for preclinical drug and biomaterial testing

Authors

Sara Svanberg[1], Mathilde Hanoune[1], Thimios A. Mitsiadis[2], Petra S. Dittrich[1*]

Affiliations

S. Svanberg and P. S. Dittrich[*]

[1]Department of Biosystems Science and Engineering, ETH Zurich, Switzerland

T. A. Mitsiadis

[2] Institute of Oral Biology, University of Zurich, Switzerland

[*]Address corresponding author:
Petra S. Dittrich,
ETH Zürich
Schanzenstrasse 44
CH-4058 Basel/Switzerland

e-mail: petra.dittrich@bsse.ethz.ch

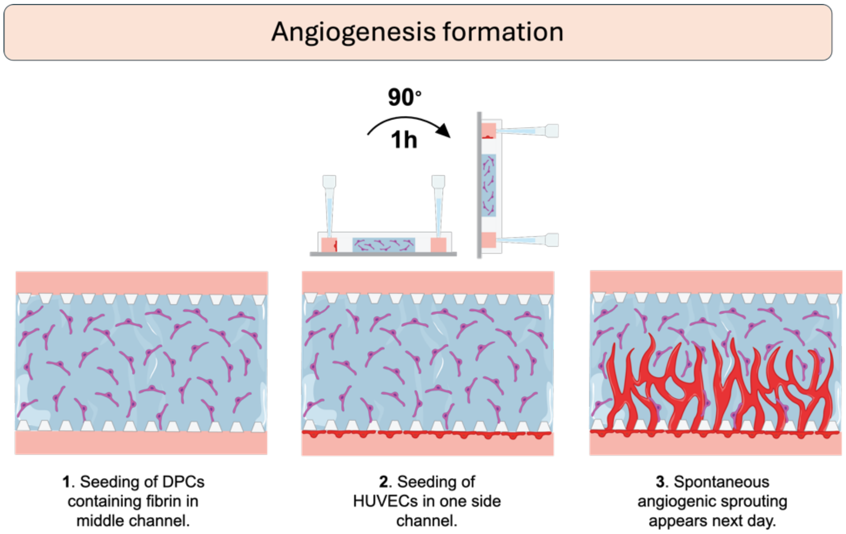

SI figure 1. Schematic sketch of seeding procedure for angiogenesis formation.

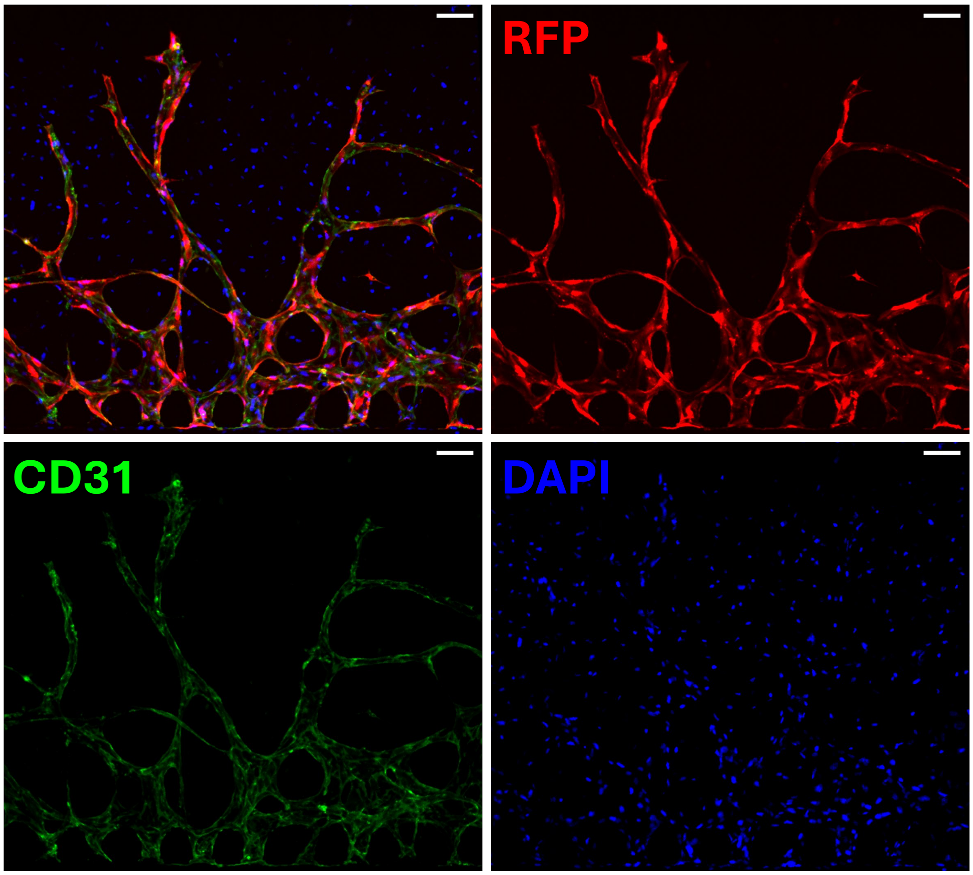

SI Figure 2. Angiogenic sprouts from RFP-producing HUVECs stained with CD31 and DAPI. Scale bars: 100 µm.

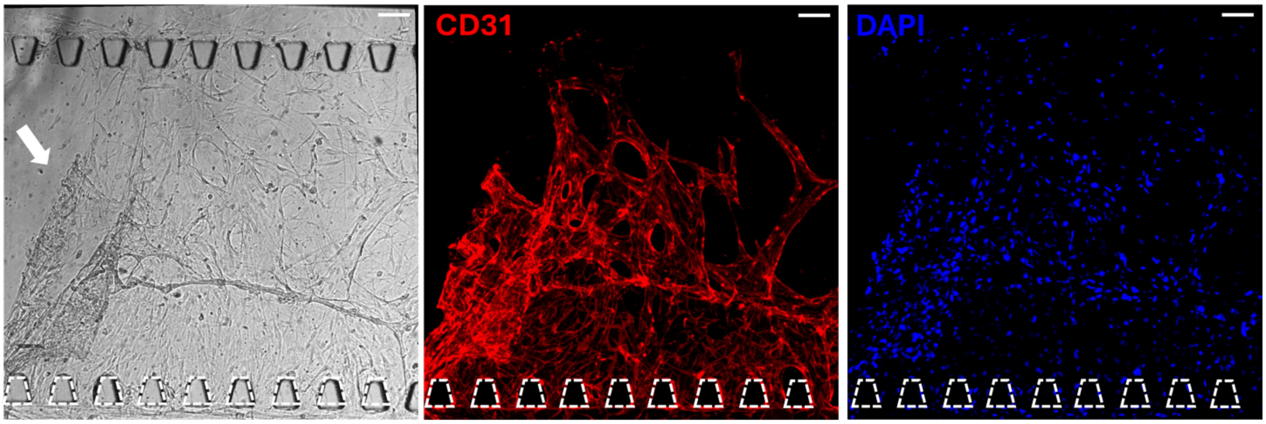

SI figure 3. Fibrin hydrogel 1.5 mg/ml fixed and stained on day 6. Brightfield (left), CD31 (red, middle) and dapi (blue, right). White arrow indicates where the fibrin hydrogel has detached. PDMS pillars at the HUVECs monolayer are indicated with dashed lines in white. Scale bars: 100 μm.

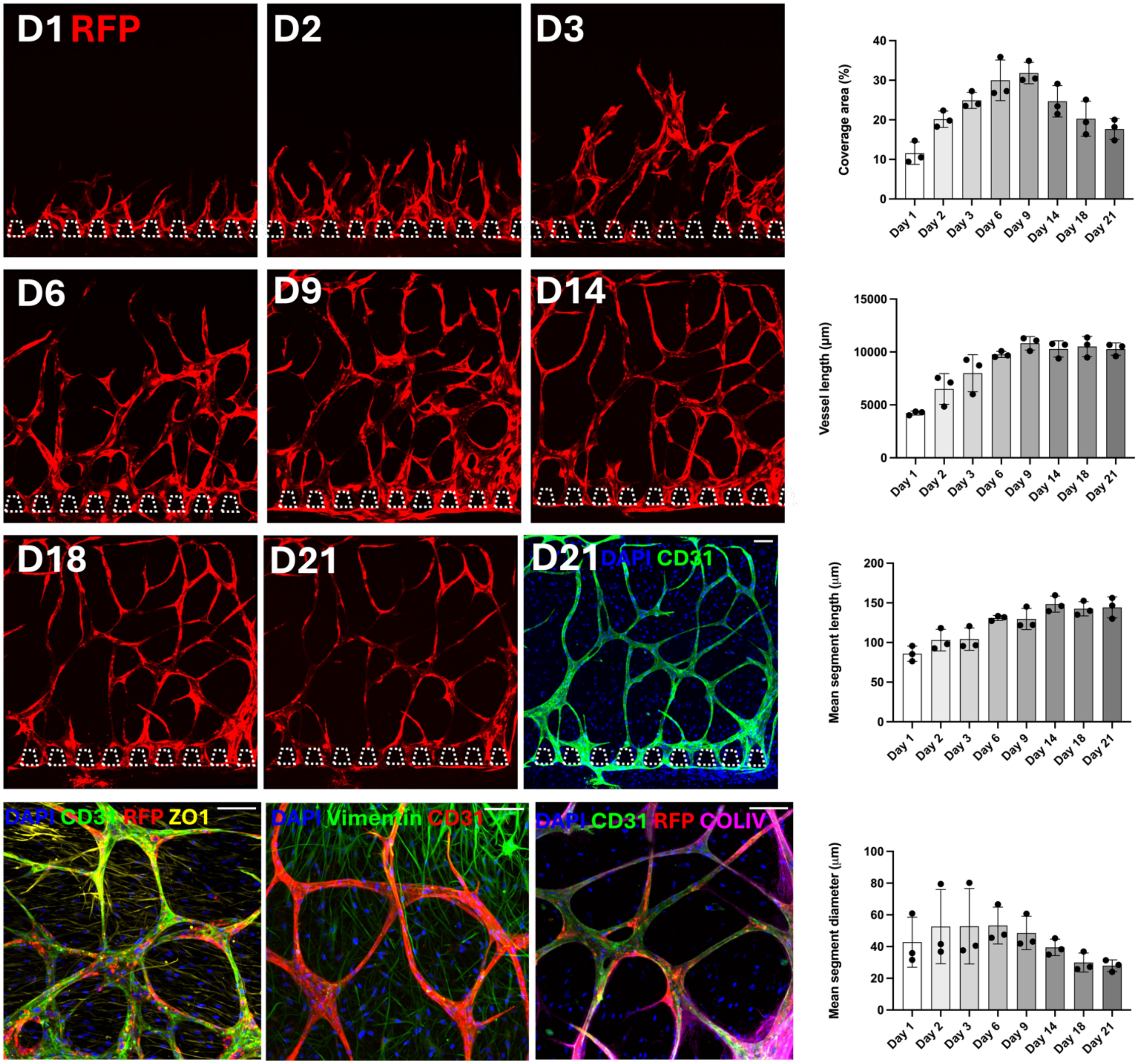

SI Figure 4. Evolution of growing angiogenic sprouts was monitored on chip over 21 days. D1-D21 show maximum projections of RFP- producing HUVECs from confocal stacks. On day 21 the cells were fixed and stained for markers including (CD31, ZO1, Vimentin and Collagen IV). The angiogenic sprouting is additionally quantified through coverage area (%), total vessel length (µm), mean segment length (µm) and mean segment diameter (µm). Each data point represents one chip analysed for three distinct regions.

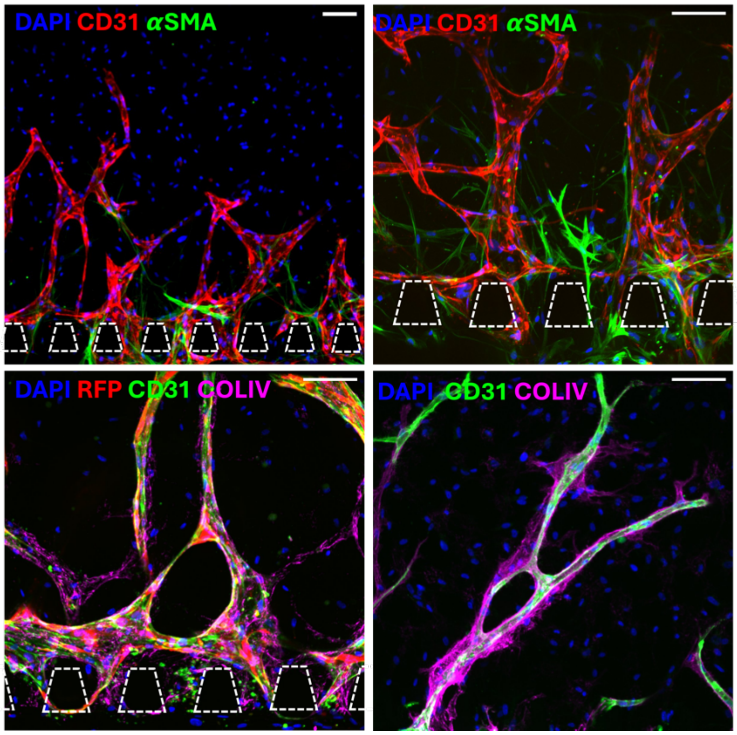

SI Figure 5. Angiogenic sprouts on day 6 during optimized culture conditions stained for CD31, αSMA and Collagen IV. Maximum projections of confocal stacks with 10X or 20X magnification. PDMS pillars are indicated with dashed lines (white). Scale bars: 100 µm.

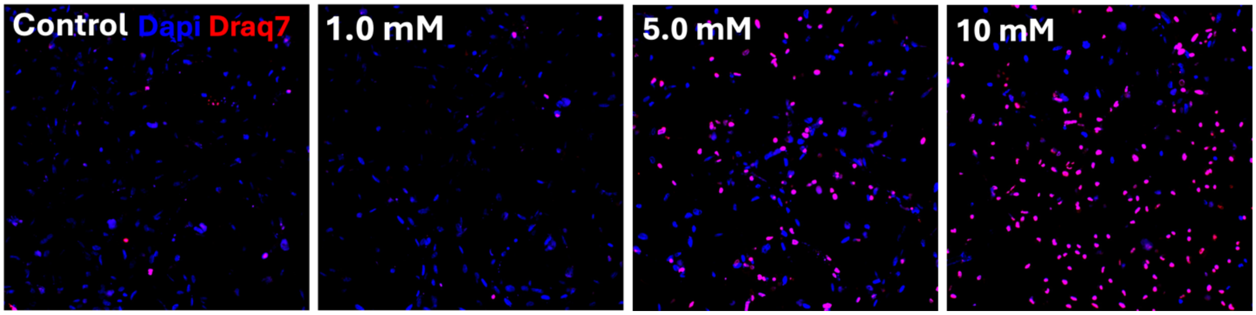

SI Figure 6. Maximum intensity projections of confocal stacks. The cells have been treated with HEMA (1.0 mM, 5.0 mM and 10 mM) and were stained with Dapi (blue) and Draq7 (red) followed by live imaging at 20X.

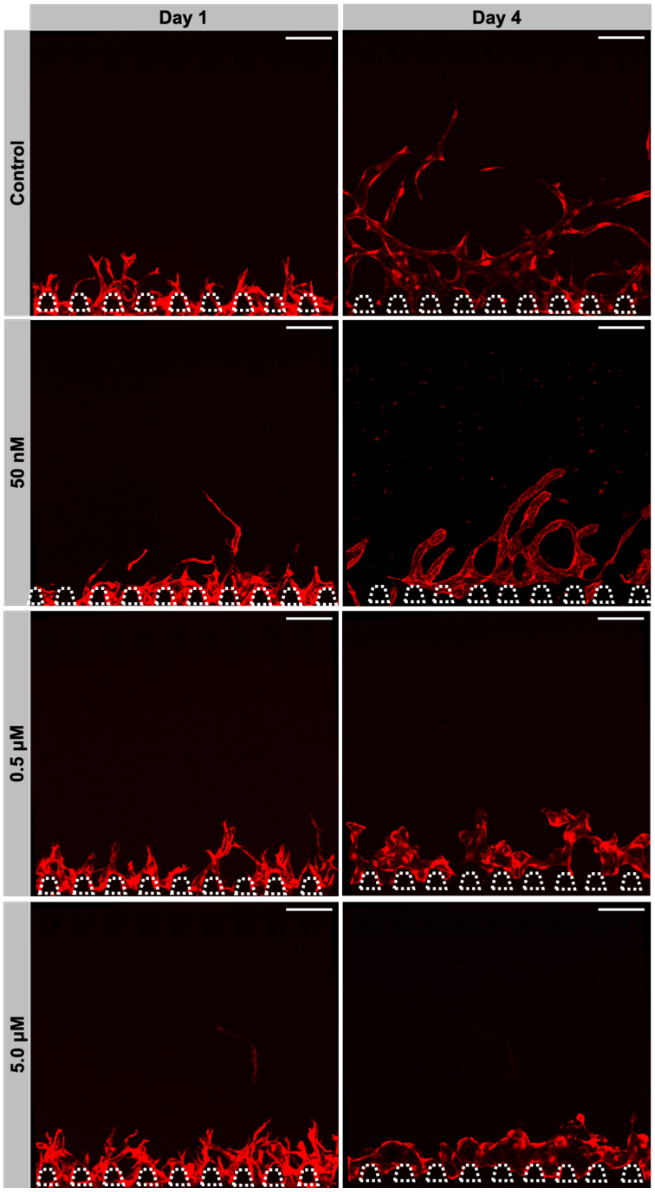

SI Figure 7. Maximum projections of confocal images. RFP-producing HUVECs (red) on chip were treated with Paclitaxel (50 nM, 0.5 µM and 5.0 µM). PDMS pillars are indicated with dashed lines (white). Scale bars: 200 µm.

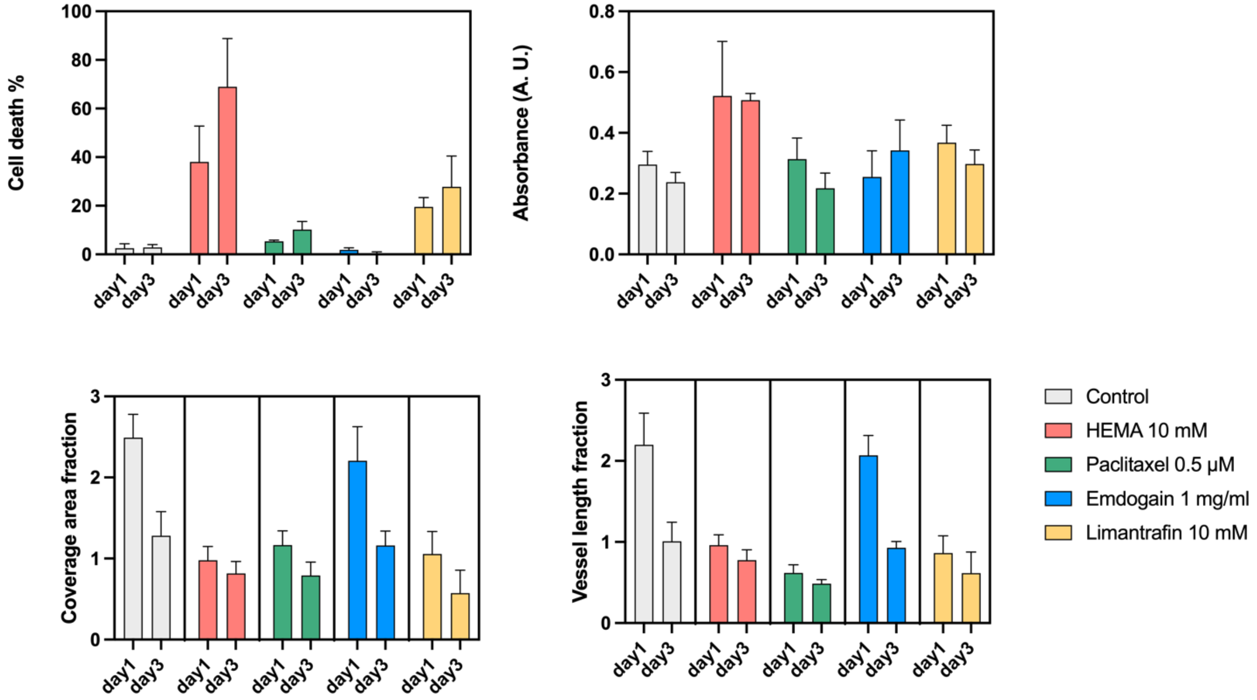

SI Figure 8. Comparison of cell death (%), LDH release and fold increase in coverage area fraction and total vessel length fraction from day 1 and day 3 treatments.
